# *In utero* Therapy for the Treatment of Sickle Cell Disease: Taking Advantage of the Fetal Immune System

**DOI:** 10.3389/fcell.2020.624477

**Published:** 2021-01-22

**Authors:** Alba Saenz de Villaverde Cortabarria, Laura Makhoul, John Strouboulis, Giovanna Lombardi, Eugene Oteng-Ntim, Panicos Shangaris

**Affiliations:** ^1^College of Medicine and Veterinary Science, The University of Edinburgh, Edinburgh, United Kingdom; ^2^GKT School of Medical Education, King's College London, London, United Kingdom; ^3^School of Cancer & Pharmaceutical Sciences, Kings College London, London, United Kingdom; ^4^School of Immunology & Microbial Sciences, King's College London, London, United Kingdom; ^5^School of Life Course Sciences, Kings College London, London, United Kingdom

**Keywords:** prenatal therapy, *in utero* transplantation, sickle cell disease, tregs, congenital blood disorders, haematopoietic stem cell transplantation (HSCT)

## Abstract

Sickle Cell Disease (SCD) is an autosomal recessive disorder resulting from a β-globin gene missense mutation and is among the most prevalent severe monogenic disorders worldwide. Haematopoietic stem cell transplantation remains the only curative option for the disease, as most management options focus solely on symptom control. Progress in prenatal diagnosis and fetal therapeutic intervention raises the possibility of *in utero* treatment. SCD can be diagnosed prenatally in high-risk patients using chorionic villus sampling. Among the possible prenatal treatments, *in utero* stem cell transplantation (IUSCT) shows the most promise. IUSCT is a non-myeloablative, non-immunosuppressive alternative conferring various unique advantages and may also offer safer postnatal management. Fetal immunologic immaturity could allow engraftment of allogeneic cells before fetal immune system maturation, donor-specific tolerance and lifelong chimerism. In this review, we will discuss SCD, screening and current treatments. We will present the therapeutic rationale for IUSCT, examine the early experimental work and initial human experience, as well as consider primary barriers of clinically implementing IUSCT and the promising approaches to address them.

## Introduction

**Figure 1:**
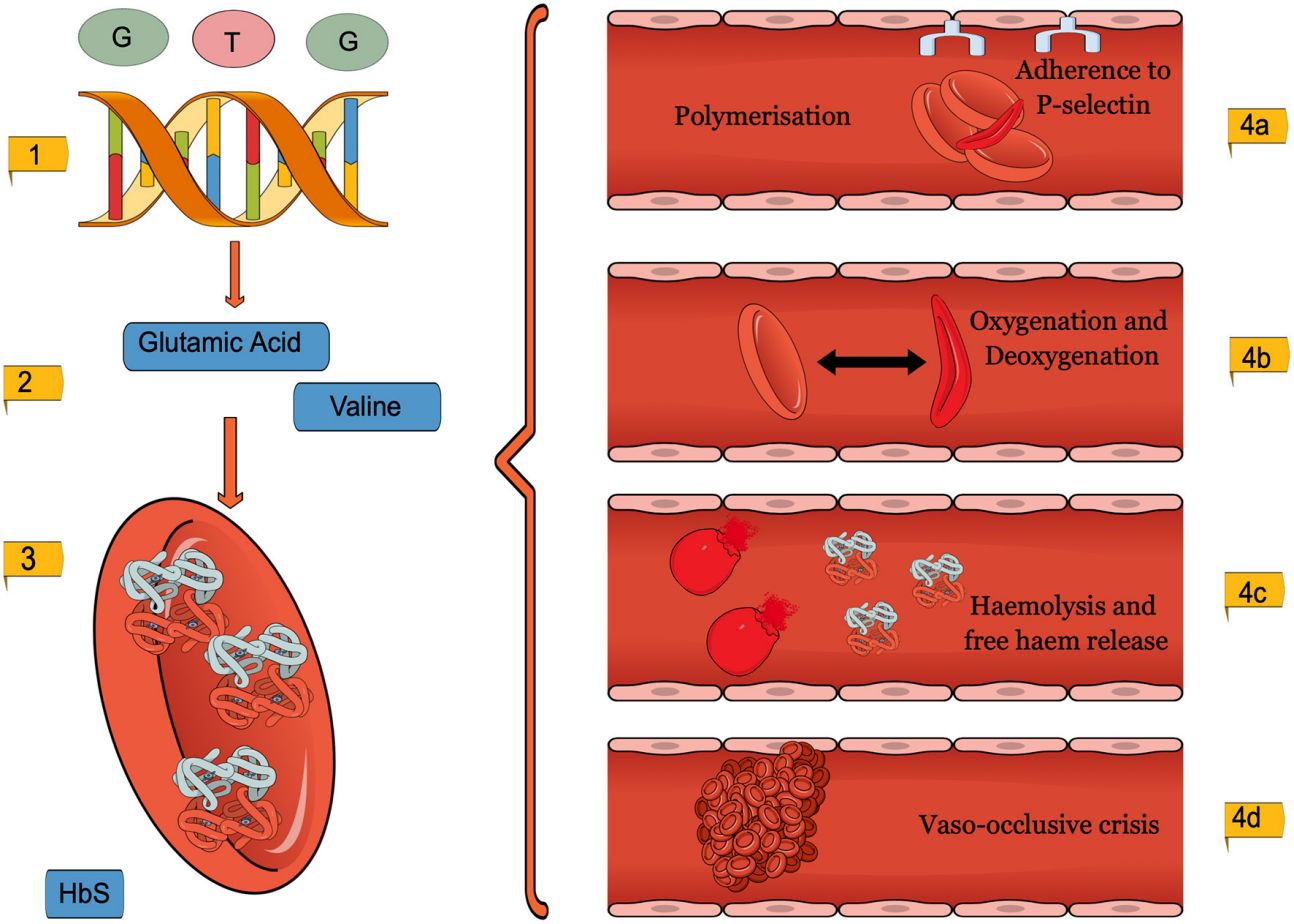
A diagram to show the pathophysiology of SCD. (1). GAG to GTG missense mutation (2). Amino acid substitution from Glutamic acid to Valine. (3). Morphology changes in the SCD erythrocytes (4). The adverse consequences, micro and macrovascular complications of SCD erythrocytes. This figure was created using MindtheGraph.com under a Creative Commons license.

Sickle Cell Disease (SCD) is an autosomal recessive disorder resulting from a β-globin gene missense mutation (Figure 1). The “sickle” hemoglobin HbS is prone to polymerisation, thereby changing erythrocyte morphology, and inducing subsequent haemolytic anemia and vaso-occlusive crises (VOCs). The primary management strategy of SCD remains focused on symptom control, and despite significant progress in understanding the condition, haematopoietic stem cell transplantation (HSCT) remains the only curative option (Lucarelli et al., 2012). Advancement in prenatal diagnosis and fetal therapeutic intervention has increased the possibility of *in utero* management, drastically changing the paradigm for SCD treatment before birth. Indeed, this life-long, debilitating condition can be diagnosed prenatally, using traditional invasive procedures such as chorionic villus sampling in high-risk heterozygous patients or fetal DNA quantification

from maternal blood (Daniel et al., 2019). Among the possible prenatal treatments, *in utero* stem cell transplantation (IUSCT) shows the most promise (Jeanblanc et al., 2014). IUSCT is a non-immunosuppressive alternative conferring various fetally focused advantages over postnatal stem cell management. Fetal immunologic immaturity may allow engraftment of allogeneic cells before fetal immune system maturation, enhancing donor-specific tolerance and lifelong chimerism (Flake, 2004). Despite host cell competition within the fetal and maternal immune systems, as well as practical aspects of IUSCT, progress is being made—preclinical studies are underway to overcome these barriers and achieve successful clinical implementation.

### Haemoglobinopathies

Sickle cell disease is part of a group of diseases called haemoglobinopathies. The severity of these diseases can vary from fatal to asymptomatic and result from structural abnormalities of the globin protein, thereby affecting erythrocytes and oxygen transport. SCD is one of the most common inherited diseases caused by a single base-pair point mutation. There is an estimated 5% global prevalence of healthy gene carriers of SCD or thalassemia as reported by the World Health Organization (Inusa et al., 2019; Sickle Cell Disease|WHO Regional Office for Africa, 2020).

### Hemoglobin Development

Hemoglobin is formed by four different globin subunits; the combination of erythrocytes and reticulocytes varies depending on the age of the individual (Inusa et al., 2019). Up to 6 weeks post-birth, fetal hemoglobin (HbF) is formed by two alpha and two gamma globin chains, coded by gene loci on chromosomes 16 and 11, respectively (Sankaran and Nathan, 2010). At 6-weeks post-partum, erythrocyte progenitors begin to produce adult (HbA) hemoglobin (Sankaran and Nathan, 2010; Diepstraten and Hart, 2019). Unlike HbF, HbA is made of two alpha and two beta globin chains and typically forms 90–95% of the total hemoglobin in adult erythrocytes, although this is subject to variation (Wood et al., 1975; Kato et al., 2018).

## Cause of Disease

### Sickle Cell Disease

SCD is the name given to a group of disorders, which contain at least one hemoglobin S allele (HbS). The second pathogenic variant can be another hemoglobin S allele or other Hb variants such as HbC (HBB GLU6LYS). The allele is caused when the Adenine base replaces Thymine in a missense mutation at the 6th position of the β-globin chain, resulting in a GAG to GTG codon change (Figure 1) (Ingram, 1957, 2004; Lettre and Bauer, 2016). SCD can be inherited as homozygous variants of hemoglobin S allele (HbSS) (Neel, 1949; Inusa et al., 2019; Mohammed-Nafi'u et al., 2020), resulting in a severe form of the disease labeled sickle cell anemia (SCA) or by compound heterozygous inheritance (HbSC). Co-inheritance of beta-thalassemia, resulting in low globin protein production, can result in HbS/B+ or HbS/Bo genotypes (Steinberg and Sebastiani, 2012; Inusa et al., 2019). Environmental factors, such as healthcare access, socioeconomic status, nutrition, and even humidity, are also known to affect disease severity (Jones et al., 2005; Tewari et al., 2015; Kato et al., 2018).

### Incidence, Burden, and Screening

Individuals of African, Indian or Arabian ancestry have a disproportionately higher prevalence of SCD. There is a theoretical advantage provided by heterozygous SCD inheritance, as carriers of SCD are 90% less likely to suffer from severe malaria (Allison, 1954; Weatherall, 2011; Luzzatto, 2012). In Africa, SCD is estimated to account for 9–15% of all deaths of children under five (Weatherall, 2010). In comparison, even with the severe form of the disease, SCA patients have a life expectancy in the UK of 67 years, with 94% childhood survival rates for uncomplicated SCD (Quinn et al., 2010; Gardner et al., 2016). Migration has distributed SCD worldwide, highlighting healthcare inequalities, where the transition from acute to chronic settings remains limited to high-income countries (Piel et al., 2013b; Lindenau et al., 2016).

In 2006, the World Health Organization recognized SCD as a global problem with significant economic implications with the incidence expected to rise above 400,000 births per year in 2050 (Piel et al., 2013a; Thein and Thein, 2016). In the UK, prenatal screening for SCD is performed in high-risk pregnancies at around 11–13 weeks, through chorionic villus sampling. A potential new non-invasive technique, which acquires fetal DNA from maternal circulation, could be the key to a safer, and potentially earlier diagnosis at 10 weeks of gestation (Lewis et al., 2014; Twiss et al., 2014; Daniel et al., 2019).

### Pathophysiology

SCD and its complications are a result of erythrocyte and reticulocyte morphology changes, seen in Figure 1 (Inusa et al., 2019; Carden et al., 2020). The mutation causes Glutamic acid to be replaced by the amino acid Valine, which changes the shape and physical properties of hemoglobin (Inusa et al., 2019). The SCD erythrocytes are then subject to intracellular HbS polymerisation (Pawliuk et al., 2001; Vekilov, 2007). Whilst healthy hemoglobin reversibly rearranges during deoxygenation, sickle hemoglobin, on the other hand, forms helical structures (Kuypers, 2014; Sundd et al., 2019). The rigid, insoluble strands form fibers which align in parallel, creating intracellular crystals (Inusa et al., 2019). At first, sickling is reversible, yet repetitive cycles of oxygenation and deoxygenation eventually lead to the characteristic irreversibly sickle cell (Noguchi and Schechter, 1985; Nash et al., 1988; Harrington et al., 1997; Goodman, 2004).

HbS polymerisation results in rigid, fragile sickle cells with up to a 75% reduction in lifespan (Kaul et al., 1983; Evans and Mohandas, 1987; Quinn et al., 2016). While normal erythrocytes live 120 days, sickle erythrocytes typically last only 10–20 days due to the processes of extravascular phagocytosis and intravascular haemolysis (Crosby, 1955; Sebastiani et al., 2007).

Oxidative stress is both cause and effect of increased erythrocyte haemolysis (Kato et al., 2018). It is triggered by HbS polymerisation and cumulative impacts of reactive oxygen species and free radicals, produced by intracellular hemoglobin metabolism (Jagadeeswaran and Rivers, 2017; Alayash, 2018). Haemolysis leads to higher levels of nitric oxide consumption, disruption of arginine metabolism, depletion of endogenous glutathione and increased expression of oxidases such as xanthine dehydrogenase (Aslan and Freeman, 2007; Wood and Granger, 2007). High levels of oxidative stress also increase damage to cell membranes. The oxidation of cytoskeletal proteins and membrane lipids prematurely ages sickle cells, further increasing haemolysis (Rogers et al., 2013).

Haemolysis releases hemoglobin and haem into the bloodstream (Kato et al., 2018). The presence of free hemoglobin and haem exacerbates extracellular oxidative stress, leading to a higher attraction between HbS molecules, agglutination and polymer formation (Kuross et al., 1988; Uzunova et al., 2010; Alayash, 2018). Free plasma hemoglobin activates both the innate immune system, through Damage-Associated Molecular Patterns (DAMPs) and platelets, through P-selectin cell surface expression (Kato et al., 2018).

SCD erythrocytes contain dysfunctional membrane channels, such as K-Cl cotransporters or Gardos channels, which contribute to cell dehydration, further increasing the HbS concentration and polymerisation (Frenette and Atweh, 2007; Gardner, 2018). Altered morphology increases membrane micro vesiculation and the release of erythrocyte microparticles, containing cell surface markers, which affect coagulation, inflammation and endothelial adhesion (Piccin et al., 2007; Westerman et al., 2008; Hebbel and Key, 2016). The resulting endothelial dysfunction is thought to involve other adhesion molecules, such as integrins, blood group antigens and white cell proteins (Takehara et al., 1991; Telen, 2005; Chen et al., 2016; Koehl et al., 2017).

### Complications

**Figure 2:**
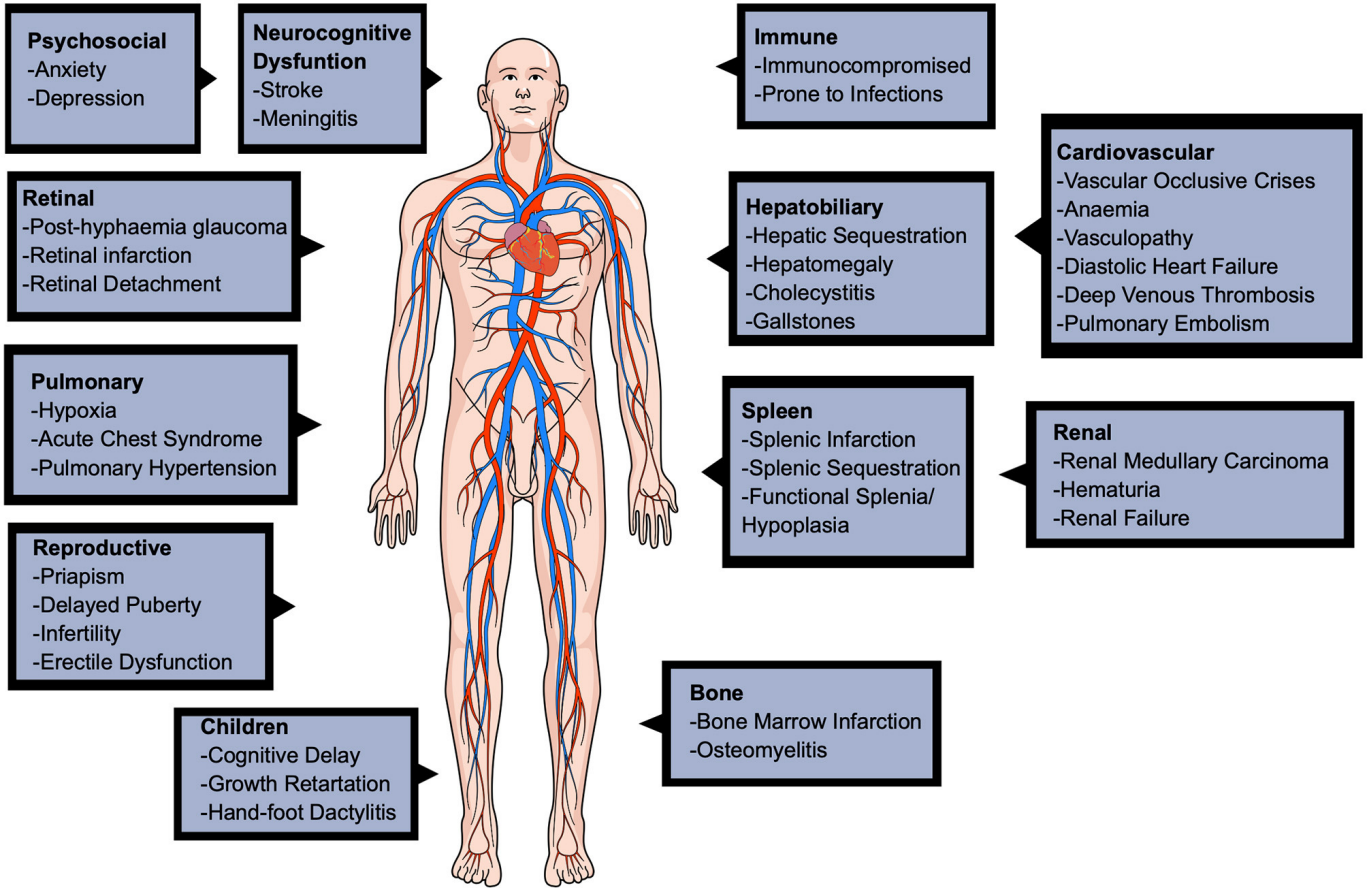
A diagram to show the various complications of sickle cell disease. The complications are seen in many major organ systems, such as cardiovascular, pulmonary, renal, hepatobiliary, reproductive and even neurological. This figure was created using MindtheGraph.com under a Creative Commons license.

SCD causes a wide range of complications in various of the body's systems, shown in Figure 2, including the cardiovascular, reproductive, pulmonary, renal, hepatobiliary, and can even cause neurocognitive dysfunction (Kato et al., 2018; Inusa et al., 2019). Beyond physical complications, SCD significantly affects the patients' quality of life, leading to psychosocial implications in infancy, adulthood, and pregnancy (Oteng-Ntim et al., 2015).

The clinical hallmark and primary cause of SCD hospitalisations are vaso-occlusive crises (VOCs) (Rees et al., 2010; Manwani and Frenette, 2013). VOCs are sudden, episodic, severe pains in the extremities, chest and back that significantly impact the patients' quality of life (de Montalembert, 2002). The painful episodes are unpredictable, spontaneous and of variable duration, generally triggered by a range of stressors such as infection, fever, dehydration, acidosis, and temperature or humidity changes. VOCs are a result of microvascular occlusions, leading to ischaemia, oedema, pain, through stimulation of nociceptive nerve fibers and papillary necrosis, eventually leading to multi-organ damage. In infants, VOCs present as irritability and inability to weight-bear, walk or crawl (Inusa et al., 2019).

## Disease-Modifying Drugs

### Hydroxyurea

Hydroxyurea acts as an inhibitor of ribonucleotide reductase, thereby inactivating enzymes involved in cellular division (Inusa et al., 2019). The result is an intermittent cytotoxic suppression of erythroid progenitors and cell stress signaling, due to cell cycle arrest and death at S phase (Agrawal et al., 2014). The resulting increased recruitment of erythroid progenitor cells (which can produce red blood cells) is associated with an overall higher HbF synthesis (Wood et al., 1976; Agrawal et al., 2014). Higher levels of HbF increase SCD lifespan, reduce the incidence of acute chest syndrome and occurrence of VOCs, stroke and chronic organ damage (Ballas et al., 1989; Strouse and Heeney, 2012; Diepstraten and Hart, 2019; Lagunju et al., 2019).

Hydroxyurea has been shown to improve both clinical and hematological complications, with particular benefit to high-risk children (Platt et al., 1984; Ballas et al., 1989; Mvalo et al., 2019). However, effectiveness varies among individuals. Due to its mechanism of action and teratogenic effect, it is contraindicated in acute liver diseases and pregnancy (Depass and Weaver, 1982; Agrawal et al., 2014; Husain et al., 2017). Adverse effects, although transient and dose-dependent, include severe anemia (1%), transient gastrointestinal symptoms, abdominal pain, vomiting, diarrhea, skin, palm and nail hyperpigmentation and short-lived hematological toxicity, with potential leukopenia, thrombocytopenia, and pancytopenia (Ballas et al., 1989; Wang et al., 2011).

Although 63% of SCA patients in high-income countries receive hydroxyurea, due to lack of treatment guidelines, reduced clinical infrastructure and medication inaccessibility, this percentage can be as low as zero in under-resourced settings (McGann et al., 2016; Power-Hays and Ware, 2020). Treatment is also burdened by poor medication adherence, high patient discontinuation, or qualitative HbF level assessments every 3–6 months (Agrawal et al., 2014; Shah et al., 2019).

### L-Glutamine

Glutamine, in addition to 5 other amino acids, is a “conditionally essential” amino acid for the body, meaning its synthesis is limited under specific physiological conditions (Inusa et al., 2019). It is thought to contribute to the production of nicotinamide adenine dinucleotide (NAD), a ubiquitous redox cofactor in red blood cells (RBC), which is reduced in SCD erythrocytes. The reduced redox potential of SCD erythrocytes increases oxidative stress, thus potentiating SCD pathophysiology (Niihara et al., 2018). In 2017, L-glutamine was approved for use in adults and children above five (Quinn, 2018; Sadaf and Quinn, 2020). Dosing is determined through weight-based formulation and given by oral administration, either in capsule, powder, or tablets (Mok and Hankard, 2011). However, hepatic and renal function monitoring is recommended (Niihara et al., 2018; Cox et al., 2020). Moreover, L-glutamine is up to 20 times more expensive than hydroxyurea and lacks long-term follow-up studies (Quinn, 2018). It requires dosage twice a day and therefore poses further feasibility, accessibility and adherence issues compared with hydroxyurea (Langley, 2020).

### New Therapeutic Approaches

Increased knowledge regarding SCD pathophysiology has led to new therapeutic strategies. These focus on fetal hemoglobin induction, HbS polymerisation and oxidation inhibition and reduced endothelial activation (Inusa et al., 2019). However, current SCD management using hydroxyurea, L-glutamine or other disease-modifying drugs is only aimed at delaying, preventing, and mitigating complications. The introduction of curative therapies, such as IUSCT has given new hope, not only for the management of SCD but for its eradication.

## Iusct for SCD

### Rationale for IUSCT

IUSCT is an appealing approach to SCD treatment as it tends to avoid the many complications associated with current prescribed management. The only viable curative treatment option for SCD is HSCT. There are various ontogenetic advantages unique to fetal development, which provide convincing arguments as to why HSCT would be far more effective if administered in the fetus rather than postnatally. Firstly, the undeveloped fetal immune system allows for the introduction of an antigen without immunosuppression and results in antigen-specific immune tolerance (Witt et al., 2018). IUSCT evades the postnatal HSCT complications such as myeloablative preconditioning, graft-vs.-host disease, and graft rejection (Witt et al., 2018). In humans, this immunological immaturity tends to be present until the end of the first trimester and so provides a succinct “window of opportunity.” Another potential biological advantage is that the early gestational period is the only time in life during which large-scale migration of hematopoietic stem cells (HSCs) to tissue compartments occurs; definitive haematopoiesis begins in the yolk sac and aorta-gonad-mesonephric region, migrates to the fetal liver, and finally resides in the bone marrow (BM) (Christensen et al., 2004). Although it was previously thought that the development of these new niches would facilitate donor cell engraftment, it is now recognized that the fetal haematopoietic system is incredibly competitive with excess circulating HSCs. However, if one could understand and utilize the natural mechanisms that regulate migration and engraftment of stem cells, it may be possible to drive the engraftment of donor cells.

In addition, the physiological conditions for the systemic distribution of donor cells are superior in the fetus, due to the presence of circulatory shunts. This allows donor cells to bypass the pulmonary circulation via the ductus arteriosus and foramen ovale and avoid sequestration in the lung microcirculation. Instead, donor cells are delivered directly into the systemic circulation and to the peripheral organs (Ekblad-Nordberg et al., 2019). Furthermore, the small size of the fetus (~35 g at 12-weeks' gestation) confers a clear advantage over transplantation in a pediatric/adult patient as it allows the cell dose per weight of the recipient to be maximized, favoring successful engraftment (Almeida-Porada et al., 2016). This approach may partly facilitate overcoming the competitive advantage of host haematopoiesis.

Perhaps the most persuasive motivation for IUSCT is the possibility of pre-empting the clinical manifestations of SCD. Treating SCD early enough to prevent disease onset in adult erythropoiesis eliminates the need for lifelong, non-curative treatment and significantly improves the quality of life for both the patient and their family.

### The Fetal Immune System

Early in development, the fetal immune system undergoes the process of self-education, principally in the fetal thymus: pre-lymphocytes that recognize “self” major histocompatibility complex (MHC) antigens are positively selected for, while those with high-affinity recognition of self-antigens are eliminated (Takahama, 2006). This results in central tolerance and should leave T cells that are reactive to foreign antigens unaffected. Theoretically, the introduction of donor cells into the fetal thymic microenvironment by IUSCT should lead to the removal of alloreactive T cells and, consequently, donor-specific immunologic tolerance. In order to achieve immunologic tolerance to donor antigens, the transplant should be performed before the emergence of mature T cells in the fetal thymus and peripheral circulation (~12–14 weeks gestation in humans) (Takahama, 2006). Thymic selection is highly effective, but not infallible; self-reactive T cells can evade deletion and circulate peripherally. However, most of these self-reactive T cells do not cause any problems as they are suppressed by fetal regulatory T cells (Tregs) (Burt, 2013).

### IUSCT Experimental Studies

The notion of using the fetus as the recipient of stem cell transplantation traces back to the landmark finding that exposure to foreign antigens can result in immune tolerance. Owen (1945), observed lifelong haematopoietic cell chimerism and sibling-specific tolerance in monochorionic dizygotic bovine twins that shared cross-placental circulation (Merianos et al., 2008). Natural haematopoietic chimerism has now been observed in numerous species, including humans and primates (Picus et al., 1985; Rinkevich, 2001).

Efforts to reproduce “natural chimerism” and treat genetic disorders in the laboratory have had varying degrees of success. The first experimental IUSCT success employed transplacental injection of adult allogeneic BM haematopoietic cells at E11, into c-kit deficient mouse fetuses, which reversed the genetic anemia produced by the c-kit defect. Interestingly, they found that the donor HSCs engrafted and successfully replaced the defective erythroid lineage. This was demonstrated by using strain-specific hemoglobin markers. The degree of correction was proportional to the degree of anemia. No engraftment was observed in non-anemic, control recipients. There was tolerance toward the transplanted HSCs with no graft-vs.-host disease observed (Fleischman and Mintz, 1979). This key finding suggests that when normal cells have a competitive advantage, greater donor cell engraftment occurs. This was also the first study to identify host cell competition as a barrier to donor cell engraftment; this is essential to consider for clinical implementation of IUSCT and will be discussed later.

Murine models of SCD have been used to demonstrate stable allogeneic engraftment following IUSCT of donor BM or fetal liver cells (Hayashi et al., 2003). However, the quantity of chimerism achieved did not correct the SCD phenotype. Despite greater levels of donor Hb (13–35%) with minimal donor mononuclear cell engraftment (1.1–4%), a substantial amount of sickling cells persisted in circulation (Hayashi et al., 2003). This finding aligns with previous studies that suggest thresholds of 70 and 40% donor myeloid engraftment are necessary to eradicate peripheral erythrocyte sickling and anemia, respectively (Iannone et al., 2001). In beta thalassemia previous experience with mixed chimerism, indicates that the disease can be significantly ameliorated by levels of BM engraftment of 15–20% (Andreani et al., 1996, 2000; Hayashi et al., 2003). Even if low engraftment is achieved (<10%), the potential of these HSCs achieving complete RBC chimerism is high, as seen in postnatal studies (Fouzia et al., 2018). Extending these studies further, Peranteau et al. administered donor BM cells intraperitoneally to murine fetal SCD model on day 14 of gestation. Postnatally, the recipients had boost transplantation with the same donor BM cells (Peranteau et al., 2015). They showed that IUSCT alone allowed for the development of donor-specific tolerance and long-lasting low levels of allogeneic engraftment. After the postnatal intervention, engraftment was enhanced to high-level chimerism and near-complete Hb replacement with normal donor Hb (>90%), ultimately correcting the SCD phenotype (Peranteau et al., 2015).

### IUSCT in Clinical Practice

The initial success of IUSCT in experimental animal models was highly promising for clinical application. As yet, IUSCT has been performed on numerous human patients for various genetic disorders, notably osteogenesis imperfecta and alpha-thalassaemia (Le Blanc et al., 2005; Götherström et al., 2014; Mackenzie, 2020). These cases demonstrated that, when using the current methodology, IUSCT is not able to reach clinically significant, therapeutic levels of engraftment unless immunodeficiency or donor cell-selective advantage was present (Merianos et al., 2008). The first successful IUSCT was achieved for “bare lymphocyte syndrome,” an immunodeficiency disorder (Touraine et al., 1989). Following this breakthrough, successful correction of severe combined immunodeficiency (SCID) was reported by several groups; stable split chimerism was achieved, with the reconstitution of the T-cell lineage only, as previously observed in murine experiments (Blazar et al., 1998). With regards to SCD, a female fetus prenatally diagnosed with SCA received donor fetal liver cells at 13 weeks of gestation (equivalent to E16 in murine pregnancy), intraperitoneally. Disappointingly, cord blood at birth and peripheral blood at 8 months of age did not demonstrate engraftment of transplanted donor cells (Westgren et al., 1996). It is, however, essential to highlight that this was accomplished over two decades ago and technical expertise has improved immensely. In addition, animal studies have uncovered many barriers to engraftment, which we will discuss.

At present, the future clinical application of IUSCT will likely ensue via two possible routes:

- A single IUSCT resulting in levels of engraftment adequate to treat SCD
- IUSCT that induces donor-specific tolerance, allowing for a second same-donor (non-myeloablative, non-immunosuppressive) transplant postnatally to boost engraftment to clinically relevant levels.

It is difficult to draw conclusions based on human clinical experience due to a number of variables; a variety of donor cell sources and transplantation methodologies have been tested, including differences in gestational age at the time of transplantation, route and amount of donor cells introduced into the fetus. These inherent inconsistencies have made it impossible to determine the factors responsible for the observed poor engraftment in human clinical cases. It is therefore vital to perform well-planned preclinical research in large animal models that somewhat resemble human fetal biology to optimize IUSCT protocols before human trials. Indeed, in 2015, MacKenzie et al. produced a consensus statement describing guidelines for IUSCT clinical trials (MacKenzie et al., 2015).

### Barriers to IUSCT

Although compelling in concept, several barriers to IUSCT have been identified through subsequent clinical and animal studies.

#### Host Competition

Perhaps the most significant barrier to donor cell engraftment following IUSCT is host cell competition. The fetus maintains strong, highly proliferative fetal compartments; therefore, IUSCT success relies on the hypothesis that donor cells can compete effectively to achieve significant levels of chimerism. The existing fetal niche has been implicated as a barrier to engraftment after it was documented that SCID recipients experienced successful engraftment of lymphoid specific cell lines and c-kit deficient mice underwent reconstitution of both erythroid and lymphoid cell lines after IUSCT (Fleischman and Mintz, 1979; Blazar and Taylor, 1995). When donor BM cells have a competitive advantage, engraftment of just a few cells can lead to reconstitution, as was seen in c-kit deficient mice where 1–2 normal HSCs fully reconstituted the haematopoietic compartment after IUSCT (Mintz et al., 1984). Nevertheless, the reverse is also true; when host cells have an advantage, it is unlikely that donor cells proliferate and engraft, irrespective of the quantity introduced into the fetus. Mechanistically, fetal stem cells show superior long-term repopulating potential compared with adult equivalents. This can be ascribed to their rapid cycling kinetics (Mazo et al., 2011; Witt et al., 2018). Murine IUSCT models have highlighted that after intravascular delivery of significantly large doses of donor total BM cells (2 × 10^11^ cells/kg fetal weight), 100% of mice maintained long-term donor cell multilineage chimerism. However, the average level of chimerism was <10% (Peranteau et al., 2006a). This suggests that host cell competition limits the level of chimerism, but not the frequency at which it occurs.

Manipulation of the fetal environment and IUSCT methodology can confer a competitive advantage to donor BM cells. One way is to deplete host niches and “create space” for donor cell expansion. It has been assumed that “space” would be available due to the rapid development of the fetal haematopoietic compartment, although this has been increasingly challenged. Indeed, Stewart et al. showed that exposure to low-dose irradiation in mice led to high levels of donor chimerism, and 10–40 million donor total BM cells produced chimerism of 40–100% (Stewart et al., 1998). Additionally, they introduced donor cells that were either non-irradiated or irradiated with the same dose as the host. Transplantation of irradiated cells reduced engraftment seven-fold, supporting the notion that competition between donor and host cells determines whether engraftment is successful. Space could still be an important factor; conditioning regimes, which can be delivered directly to the umbilical vein (avoiding maternal circulation), using ultrasound guidance might work by providing space and is worthy of consideration (Derderian et al., 2014).

Strategies to decrease competition and improve donor engraftment *in utero* have also been investigated. Derderian et al. showed that selective *in utero* depletion of host HSCs using an antibody against the c-kit receptor (ACK2), injected directly into fetal mice on E13.5–14.5, resulted in depletion of host HSCs within the BM with minimal toxicity (Derderian et al., 2014). The antibody was cleared from serum before neonatal congenic HSCT and chimerism was higher in these pups than controls. The maternal circulating erythrocyte and leukocyte counts remained within normal levels. In addition, low levels of allogenic chimerism—induced by IUSCT—were enhanced to near-complete donor engraftment in mice by postnatal minimally myeloablative total body irradiation of the pups followed by same-donor transplantation (Peranteau et al., 2002). Furthermore, after Busulfan-conditioned transplantations, mice with <1% and >1% chimerism showed a marked improvement to 60 and 100% engraftment, respectively (Ashizuka et al., 2006).

Improving the competitiveness of the donor cells is another strategy. *In vitro* studies indicate that this can be done by decreasing cleavage of stromal-derived factor-1 (SDF-1) by CD26 inhibition. CD26 is a chemokine expressed on HSCs (Christopherson et al., 2004). CD26 cleaves the amino-terminal dipeptide from SDF-1CXCL12, producing an inactive form which prevents chemotaxis. CD26 inhibitors work by enhancing the chemotaxis of HSCs to SDF-1/CXCL12. Treatment of donor HSCs along with a CD26 inhibitor (dipeptidyl peptidase) enhanced homing and long-term engraftment of allogeneic donor cells following IUSCT (Peranteau et al., 2006b). This suggests that CD26 inhibition may be a suitable adjunct. Additionally, co-transplantation with stroma can selectively influence the expression of homing receptors and ultimately improve engraftment, as hematopoietic activity increases considerably after stromal establishment (Flake and Zanjani, 1999). Indeed, co-transplanted stromal cells expressing CD146, CXCL12, and VEGF led to improved engraftment in sheep (Mokhtari et al., 2016).

#### Immunological Barriers

The only successful clinical findings have been achieved in immunodeficiency disorders, indirectly suggesting an immunological barrier to IUSCT. Historically, the overriding contradiction to this was the lack of support for a congenic advantage to engraftment over allogeneic cells. In fact, Howson-Jan et al. found a higher incidence of engraftment using allogenic (5.2%) compared with congenic (0.7%) donor cells (Howson-Jan et al., 1993). This was a transient result since all the recipients had non-detectable donor cells at 6 weeks postnatally. However, Carrier et al. (1995) reported higher microchimerism rate in congenic (25%) as opposed to allogeneic (7%) recipients after IUSCT, but there were no differences in the incidence of organ engraftment (Carrier et al., 1995). However, the insignificant chimerism and low frequency of engraftment in these studies make interpretation problematic.

The seminal paper that demonstrated the superiority of congenic transplantation, and hence suggested the presence of an immunological barrier, came from Peranteau et al. (2006a). At E14, mouse fetuses were intravascularly transplanted with high doses of donor cells of allogeneic or congenic BM cells. Despite comparable homing and initial engraftment, by 5 weeks of age, engraftment was lost in 70% of the allogeneic recipients, while all congenic mice maintained stable long-term multilineage chimerism (Peranteau et al., 2006a). Adaptive cellular and humoral alloresponses were quantitatively higher in non-chimeric than chimeric animals, suggesting that the fetal immune response was responsible for restricting donor cell engraftment. Because all congenic and allogeneic animals maintained measurable chimerism levels 2 weeks after IUHCT, it seemed that engraftment failure was a postnatal occurrence, supporting the presence of a previously unrecognized adaptive immune barrier. Studies from Merianos et al. were crucial in explaining these findings. The group observed that, if transplanted murine pups were placed with surrogate mothers that had not received IUSCT, 100% of recipients maintained chimerism (Merianos and Tiblad, 2009). This suggests that IUSCT triggered maternal alloimmunisation, resulting in the transfer of alloantibodies to the pup via breast milk, inducing an adaptive immune response and loss of chimerism. Although the exact mechanism is unknown, these observations suggest that maternal sensitization during IUHCT in humans could pose a considerable difficulty, as transplacental alloantibody transport cannot be avoided.

In addition to alloantibodies, maternal cell microchimerism, due to maternal-fetal cell trafficking, may also be a reason for rejection in IUSCT. Bidirectional cellular trafficking occurs in normal pregnancy, with maternal microchimerism representing a small percentage of total circulating fetal immune cells (Burt, 2013). The significance of maternal microchimerism in IUSCT was initially explored by Nijagal et al. who demonstrated that murine fetuses contain a substantial amount of maternal cells at baseline, which increase considerably after IUSCT of allogenic cells (Nijagal et al., 2011a,b). Interestingly, cell composition suggested that cell trafficking is an active and selective process rather than the mechanical result of the fetal injection. A significant number of maternal T cells have been found in the fetus; a maternal T cell response was proposed as the reason for lower early engraftment rates in allogeneic IUSCT (Merianos et al., 2008). In support of this, engraftment was substantially increased in T cell-deficient mothers, but not if the graft matched maternal MHC, suggesting that transplacental trafficking of maternal allospecific T cells pose a considerable obstacle. Interestingly, no maternal cells were found within the recipient after the fetal period, making it challenging to explain engraftment loss by this mechanism.

There is ample experimental and clinical evidence in human fetuses to suggest that exposure to maternal antigens by cellular trafficking leads to the establishment of fetal Tregs (Burt, 2013). These prevent fetal T cells acting against maternal antigens. The constant, small volume of immigrating maternal cells is wholly distinct from the large bolus supplied in IUSCT that disrupts normal cell number ratios. Nonetheless, it may be a mechanism of instigating tolerance (Mold et al., 2008).

Rejection by maternal immune cells does not provide a comprehensive explanation for the patterns of graft rejection seen in animal IUSCT models. This is apparent when considering that engraftment may occur in alternating littermates exposed to the same maternal environment (Durkin et al., 2008). Additionally, the fact that the maternal immune system has been intact in all human cases who have undergone IUSCT suggests that the maternal immune response cannot be a critical contributing factor in IUSCT failure. Indeed, phase 1 clinical trials at the UCSF Fetal Treatment Center are taking advantage of the possible maternal-fetal tolerance during pregnancy and are using (Mackenzie, 2020). Instead, the role of innate immunity has been brought into question after recent studies suggested the potential of fetal NK cell-mediated immune response to allogeneic donor cells (Alhajjat et al., 2015).

Durkin et al. found that engraftment correlated with a level of initial chimerism of >1.8% (chimerism threshold) and with a donor-tolerant NK response (Durkin et al., 2008). Moreover, when NK cells were depleted from sub-threshold fetal chimeras, rejection did not occur as expected. Instead, the recipients maintained stable engraftment. When NK cells were allowed to recover and increase in numbers, the engraftment was lost (Durkin et al., 2008; Alhajjat et al., 2015). These findings were vital in confirming the involvement of the innate immune system in IUSCT engraftment rejection. By identifying the chimerism threshold, this study suggested that NK cell education is essential for success. Alhajjat et al. recently showed a mechanistic link between the induction of prenatal NK cell tolerance and trogocytosis (Alhajjat et al., 2013). Trogocytosis is a process whereby lymphocytes, conjugated to antigen-presenting cells, extract surface molecules from these cells and express them on their surface. They explain how engraftment levels of 1–2% could result in adequate exposure of donor antigens to a sufficient number of NK cells to reliably induce donor-specific tolerance (Alhajjat et al., 2013).

**Figure 3:**
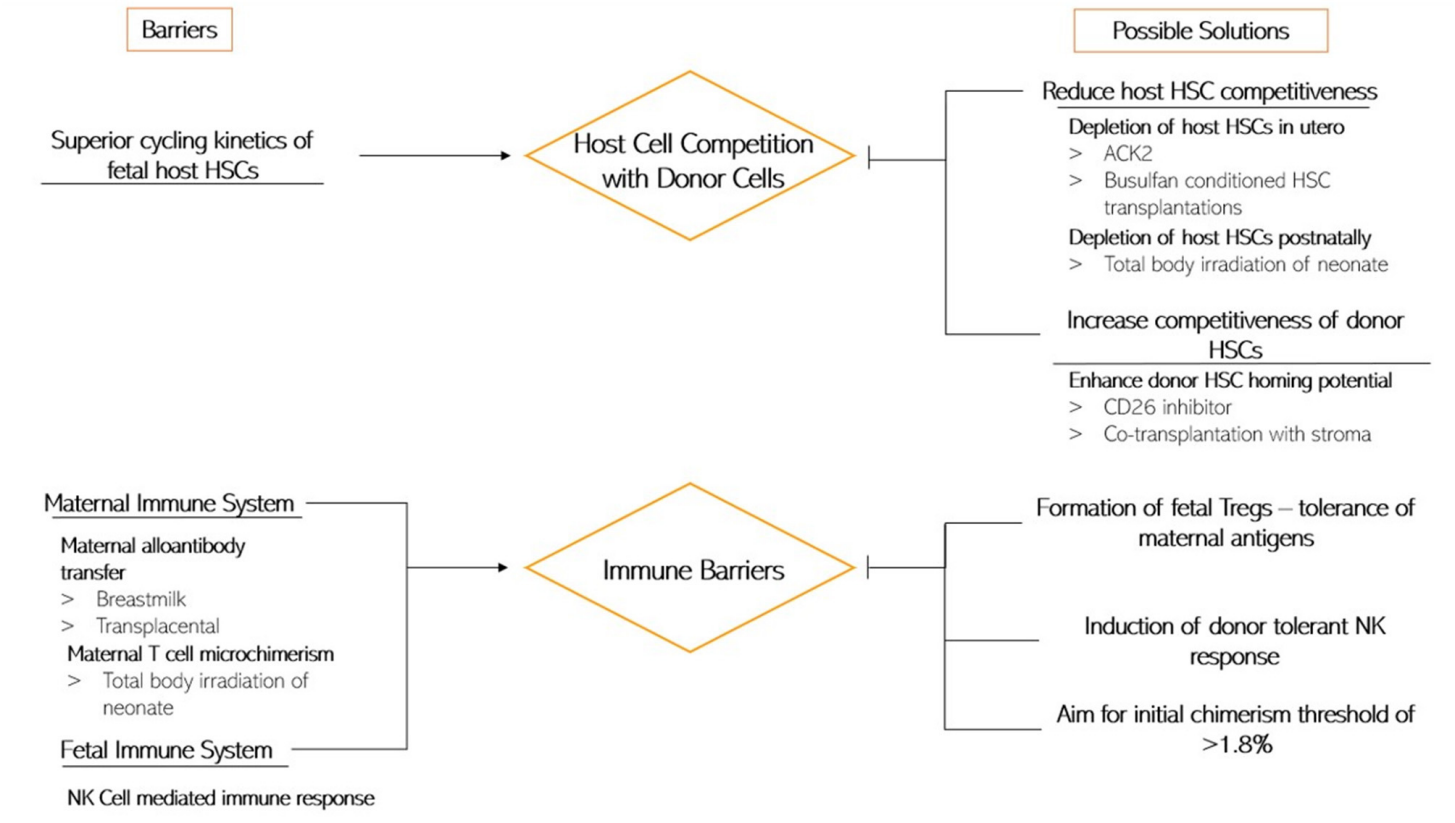
This diagram depicts the current understanding of the barriers to IUSCT **(Left)** and the possible ways of overcoming them **(Right)**.

As discussed, IUSCT has numerous fetal and maternal obstacles to overcome before it can become clinically valuable. Figure 3 summarizes the current understanding of the impediments to an application.

Another option, which bypasses some of the obstacles IUSCT has, is to deliver direct gene therapy to the fetal haematopoietic niche. For example, we could deliver gene therapy to the fetal liver, which is one of the haematopoietic organs in the early fetus. This was demonstrated by the *in-utero* injection of a lentiviral vector in a mouse model of beta-thalassaemia. This method was efficient and phenotypically corrected the heterozygous mouse model of disease (Shangaris et al., 2019). Direct gene therapy to the fetus sounds promising, even though it would raise other ethical and safety concerns which are beyond the scope of this review (Shangaris, 2020).

#### IUSCT Protocol

IUSCT is a novel course of treatment for SCD; thus patient selection and treatment protocol must be comprehensively defined. Several aspects of IUSCT have shown potential areas for improvement. As mentioned previously, the timing of this procedure is paramount to promote donor cell tolerance. In humans, the most appropriate timing for IUSCT would be during the 12th week of gestation, before the HSC colonization of the BM (Mazo et al., 2011). In addition, decisions on the source of stem cells are important. Technical improvements have highlighted that the intravascular route may be a promising new approach. Vrecenak et al. used the canine model to demonstrate that the previous method of intraperitoneal injection of donor cells was inefficient and consistently resulted in donor chimerism levels of <3%. In contrast, intravascular injections resulted in increased levels of sustained donor chimerism (>10%) (Vrecenak et al., 2014). Long-term follow-up studies have confirmed that intravascular injections are more efficient in homing to the fetal liver and intercostal vascular bundles, whereas most cells introduced via the peritoneum remained in the abdomen and were unable to migrate to haematopoietic sites. However, ovine studies have produced contradictory results, stating that the intravascular route confers no benefit (Tanaka et al., 2010). Perhaps, the safer option—intraperitoneal—may be more suitable for clinical implementation as intravascular is technically more difficult, especially at <14 weeks gestation. Infusion of donor cells into the coelomic cavity may present a future approach as it has exhibited great potential in fetal sheep models. The coelomic cavity is now believed to be an important transfer interface for the embryo, present very early in gestation (Cumano and Godin, 2007).

### Ethical Considerations

IUSCT is ultimately performed on two patients: the mother and her fetus. Ethical considerations are essential for IUSCT studies and clinical implementation. If one is to consider the fetus as a patient, possible interventions must be of tolerably low risk to the mother.

Also, as mentioned in Kregel et al. communication and sound understanding is crucial in preparing families for IUSCT (Kreger et al., 2016). Firstly, the prenatal diagnosis of SCD must be correct and reliable and needs to be explained clearly to the parents. They must be well-supported to make an informed decision about further management options. Reasonable alternatives must be offered and explained clearly. The therapeutic outcome of IUSCT is uncertain, and this must be made known, with realistic expectations clarified. It is also important to note that the availability of treatment *in utero* may impact the decision to continue or terminate the pregnancy. Therefore, provision of counseling to families considering IUSCT is vital.

## Conclusion

SCD poses a global problem with few treatment alternatives. IUSCT is non-immunosuppressive transplant approach allowing for donor cell engraftment and donor-specific tolerance in the fetus. It has the potential for treatment early on in gestation, before fetal immune system maturation. Despite promising experimental and clinical progress, there are several remaining obstacles to overcome before IUSCT is widely accepted clinically. With a greater understanding of the requirements of engraftment and the mechanisms of tolerance, strategies can be developed to achieve induction and maintenance of high or complete donor chimerism. Barriers to successful engraftment such as host cell competition, the immune systems of the fetus and mother and the methodology of IUSCT, warrant further investigation, to find new, efficacious ways of tackling them.

## Author Contributions

AC, LM, and PS conceptualized the topic and structure of the review. AC and LM drafted and revised the manuscript. PS, JS, EO-N, and GL revised and edited the draft paper. AC, LM, PS, JS, EO-N, and GL approved the final manuscript. All authors contributed to the article and approved the submitted version.

## Conflict of Interest

The authors declare that the research was conducted in the absence of any commercial or financial relationships that could be construed as a potential conflict of interest.

## Abbreviations

ACK2: C-kit Receptor
BM: Bone Marrow
DAMPs: Damage-Associated Molecular Patterns
HbA: Adult Hemoglobin
HbF: Fetal Hemoglobin
HbS: Sickle Hemoglobin
HSC: Hematopoietic Stem Cells
HSCT: Haematopoietic Stem Cell Transplantation
IUSCT: *In Utero* Stem Cell Transplantation
MHC: Major Histocompatibility Complex
SCA: Sickle Cell Anemia
SCD: Sickle Cell Disease
SDF-1: Stromal Derived Factor 1
Tregs: Regulatory T cells
VOC: Vaso-occlusive Crises.
